# Genome of the enigmatic watering-pot shell and morphological adaptations for anchoring in sediment

**DOI:** 10.1186/s12864-025-11622-w

**Published:** 2025-05-09

**Authors:** Julia D. Sigwart, Nur Leena W.S. Wong, Vanessa Liz González, Fabrizio Marcondes Machado, Carola Greve, Tilman Schell, Zeyuan Chen

**Affiliations:** 1 Senckenberg Research Institute and Natural History Museum Frankfurt, Frankfurt, Germany; 2 Institute of Ecology, Evolution & Diversity, Goethe University, Frankfurt, Germany; 3 International Institute of Aquaculture and Aquatic Sciences, Universiti Putra Malaysia, Port Dickson, Malaysia; 4 Informatics and Data Science Center, Smithsonian Institution National Museum of Natural History, Washington, DC USA; 5 Departamento de Biologia Animal, Universidade Estadual de Campinas (UNICAMP), São Paulo, Brazil; 6 LOEWE Centre for Translational Biodiversity Genomics, Frankfurt, Germany

**Keywords:** Bivalvia, Suction Caisson, Clavagelloidea, Phylogenomics

## Abstract

**Background:**

In this study, we present the first chromosome-scale genome of *Verpa penis* (Linnaeus, 1758), and the first for the bivalve clade Anomalodesmata. The present study has two separate foci. Primarily, we provide the genetic resource to bridge further studies from genome to phenome and propose hypotheses to guide future empirical investigations. Secondarily, based on morphology, we outline a conceptual exploration to address their adaptation. Watering-pot shells have been called “the weirdest bivalves” for their fused tubular shell resembling the spout of a watering can. This adventitious tube arose twice convergently in clavagelloidean bivalves. However, previous literature has never provided a convincing adaptive pathway.

**Results:**

The genome assembly of *V. penis* was about 507 Mb, with contig N50 of 5.33 Mb, and has 96.5% of sequences anchored onto 19 pseudochromosomes. Phylogenomic analyses of this new genome in context of other bivalves confirms the placement for Anomalodesmata as sister to the clade Imparidentia. Contrary to expectations from its highly modified body plan, there is no evidence of chromosome reduction compared to the ancestral karyotype of heterodont bivalves (1 *N* = 19). Drawing on established principles from engineering as well as morphology, the thought experiment about the adventitious tube seeks to extend current understanding by exploring parallels with other built structures. A new hypothesis explains one possible interpretation of the adaptive significance of this body form: it is potentially structurally optimised for vertical stability in relatively soft sediments, with parallels to the engineering principles of a suction anchor.

**Conclusions:**

While the conclusions presented here on morphological interpretations are theoretical, this serves as a foundation for further empirical validation and refinement. Our study offers new insights to a long-standing mystery in molluscan body forms and provides genomic resources that are relevant to understanding molluscan evolution, biomineralisation, and biomimetic design.

**Supplementary Information:**

The online version contains supplementary material available at 10.1186/s12864-025-11622-w.

## Background

**Fig. 1:**
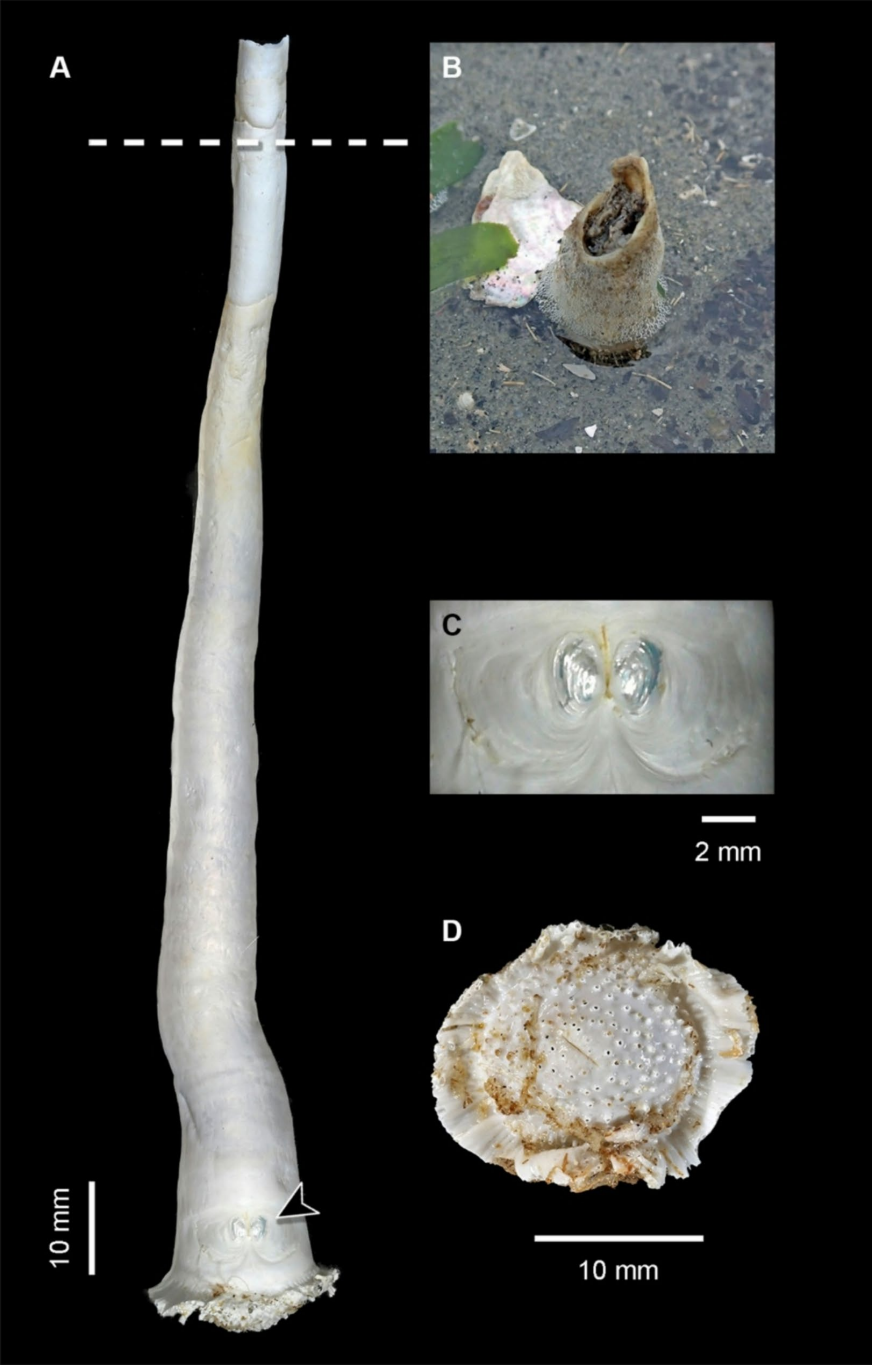
*Verpa penis*, the watering pot shell, from Merambong Shoal, Johor, Malaysia. **A**, animal in dorsal view, with dashed line indicating typical burial depth, **B**, in situ image of live animal, photo by NLWSW, **C**, close up of the larval shell (indicated by an arrowhead in **A**), **D**, anterior (bottom) “watering pot”. Photos (except **B**) of specimen SMF 367281 by Sigrid Hof

Bivalvia, a familiar and widespread molluscan clade, have provided fundamental insights for global biodiversity patterns [1, 2], but they are neither ecologically nor morphologically homogenous. The class contains frequent and repeated body plan modifications [3], such as vermiform ship worms and the mysterious watering-pot shells. For members of the superfamily Clavagelloidea (Anomalodesmata), the only resemblance to a bivalve shell is the juvenile shell retained on one (dorsal) side of the adult tube-shell. The tube widens at the base and the bottom is enclosed with a plate perforated by holes that resemble the spout or “rose” of a watering can. The siphons of the bivalve extend upward through the tube, away from the spout. At the top, only the narrow tip of the tube emerges at the sediment surface (Fig. 1).

Anomalodesmata comprises 22 living families, characterised by a wide array of highly specialised morphologies [4, 6]; they are distinctive, but often ecologically rare, and often occur in inaccessible habitats. Largely as a consequence of these confounding factors, this group is under-represented in phylogenetic analyses [5]. These highly adapted bivalves include clavagelloids and a broad group of carnivorous species that are highly diversified in the deep sea [6, 7]. The two clavagelloid families, Clavagellidae and Penicillidae, all live in shallow marine habitats but are easily distinguishable [8]. Clavagellidae retain only the left valve within the fused tube or crypt, whereas Penicillidae retain both valves; this correlates to differences in the musculature. Both families include species with endobenthic, tube-forming bodies, but also other epibenthic crypt-forming genera [8, 9], meaning that this tube has evolved twice independently [10].

The clavagelloidean body is positioned at the bottom end of the tube (anatomically anterior), with a pedal disc in contact with the perforated “watering pot”, and siphons extending vertically through the tube to reach the sediment surface. The tube forms as post-settlement growth of the shell, which expands in the normal way but at some point wraps around and fuses [8]. The term “adventitious” refers to the unusual anatomical position rather than accidental or externally moderated formation.

The adventitious tube has been described as a defensive adaptation [9, 11]. While it certainly provides armour that would protects the siphons, this hypothesis has never been scrutinised as an evolutionary driver of major morphoanatomical change. The presence of arenophilic (detritus-sticking) glands in the siphon tips of some clavagelloids [11], similar to some other anomalodesmatans [7, 12, 13] also provide camouflage that appears to have the same anti-predation function. The tubes also are frequently marked with scars of breakage from likely unsuccessful predation attempts (Fig. 1). These points indicate that the tube has some vulnerability to predation and conversely may provide protection, but this does not fully explain its parallel emergence in two families.

Formal morphocladistic analyses support the convergent evolution of this remarkable structural change [6]; however, molecular data are scarce for both families as well as any other anomalodesmatans. To date, sequence data are only available for three genera in both clavagelloid families: coral-boring *Bryopa* (Clavagellidae), including one mitogenome [4, 5, 14], and some standard markers for adventitious tube-forming *Brechites* and *Verpa* (as *Penicillus*) (Penicillidae) [4, 15]. Further data for any watering-pot shell would allow insights to the potential genomic mechanisms underlying this extreme adaptation.

## Methods

### Field collection

Animals were collected at Merambong Shoal, Johor, Malaysia, 11 February 2020 (1.3394°N, 103.6066°E). The sediment in this area is sand texture or sandy mud, characterised by a highly diverse seagrass meadow [16]. After collection, the six specimens of *Verpa penis* were kept alive in laboratory culture at I-AQUAS, Universiti Putra Malaysia, Port Dickson, for over two months (early February to late April 2020). One female individual was dissected into separate organs, with the foot and siphons preserved in ethanol and other organs preserved in RNAlater, this individual was used for all aspects of genome assembly (PacBio, Hi-C, RNAseq). The remaining specimens from the same collecting event are retained in the Universiti Putra Malaysia Marine Collection (UPMMC), Port Dickson.

### Sequencing

High molecular weight (HMW) gDNA was extracted from ethanol-preserved tissues [17] with a pre-wash step with sorbitol. DNA concentration and DNA fragment length were assessed using the Qubit dsDNA BR Assay kit on the Qubit Fluorometer (Thermo Fisher Scientific) and the Genomic DNA Screen Tape on the Agilent 2200 (Agilent Technologies). We prepared one low-input PacBio HiFi library according to the SMRTbell Express Prep Kit v2.0 instructions. To remove smaller fragments, the DNA library was size selected using beads with a cut-off at 3 kb. The same library was loaded on two SMRT 8 M cells and sequenced in CCS mode using the PacBio Sequel II instrument. The on-plate concentration was 80 pM using adaptive loading and the Sequel II Binding kit 2.2 (Pacific Biosciences, Menlo Park, CA). Pre-extension time was 2 h, run time was 30 h. HiFi reads were called using a pipeline running PacBio tools: ccs 6.4.0, actc 0.3.1, samtools 1.15 [18], and DeepConsensus 1.2.0 [19].

To prepare a chromatin conformation capture library, we used the Arima High Coverage Hi-C Kit v01 (Arima Genomics) according to the Animal Tissue User Guide for proximity ligation using approximately 44 mg of muscle tissue. The proximally-ligated DNA was then converted into an Arima High Coverage HiC library according to the Swift Biosciences Accel-NGS 2 S Plus DNA Library Kit protocol. The fragment size distribution and concentration of the Arima High Coverage HiC library were assessed using the TapeStation 2200 (Agilent Technologies) and the Qubit Fluorometer and Qubit dsDNA HS reagents Assay kit (Thermo Fisher Scientific, Waltham, MA), respectively. The library was sequenced on the NovaSeq 6000 platform at Novogene (UK) using a 150 paired-end sequencing strategy, resulting in an output of 21 Gb.

Total RNA was isolated from pedal disc, mantle, siphon and pericardium tissues using TRI-Reagenz^®^ (Sigma-Aldrich) according to the manufacturer’s instructions. The quality and concentration of each extraction were assessed using the TapeStation 2200 (Agilent Technologies) and the Qubit Fluorometer with the RNA BR Reagents Assay Kit (Thermo Fisher Scientific, Waltham, MA). The RNA extractions were then pooled at equal concentrations and sent to Novogene (UK) for Illumina paired-end 150 bp RNA-seq of a cDNA library (insert size: 350 bp) with an expected output of 10 Gb.

### Genome size Estimation

Genome size and heterozygosity were estimated from a k-mer profile of the HiFi reads (Electronic Supplementary Materials Fig S1). First, a count from Jellyfish 2.3.0 [20] was run with the additional parameters “-m 21 -s 100 M” and all HiFi reads as input, then the GenomeScope v1.0 and v2.0 [21] in combination with R 4.3.1 was executed with the k-mer size set to 21.

### Genome assembly

All the HiFi reads were processed for *de novo* genome assembly using hifiasm v0.14-r312 with default parameters [22]. To remove haplotypic duplications and overlaps in the assembly, Purge_Dups v.1.2.5 was used [23]. The Hi-C reads were then aligned to the initial genome assembly using Arima Genomics’ mapping pipeline (https://github.com/ArimaGenomics/mapping_pipeline). Reads were mapped to the reference using BWA-MEM v0.7.17-r1188 [24], converted to a sorted.bam file, and filtered to keep uniquely mapping pairs. PCR duplicates were removed using Picard v3.0.0 [25]. The final alignment file and the assembly were then passed to YaHS v 1.1a-r3 [26] for scaffolding in the default mode. Juicebox Assembly Tools [27] were used to generate and visualise a HiC contact map. We manually curated the scaffolded assembly using an editable Hi-C heatmap to improve the assembly quality and to correct misassembles with Juicebox v1.11.08 [28] (ESM Fig S2).

Genome completeness was assessed by BUSCO v5.4.3, in euk_genome_met mode [29] using the lineage dataset metazoa_odb10 (Creation date: 2021-02-17, number of genomes: 65, number of BUSCOs: 954). (ESM Tables S1-S2). Completeness regarding k-mers and QV values were obtained with Meryl 1.3 and Merqury 1.3 [30].

### Genome annotation

TRF v4.09 [31] was used for tandem repeats identification. Transposable elements (TEs) were annotated using a combination of ab initio and homology-based approaches. First, repeat elements were identified *de novo* using RepeatModeler v2.0.4 [32]. The predicted models, together with a repeat database Dfam_3.0, were then merged together and used as a custom library for RepeatMasker v4.1.5 [32] to localise, identify and mask repeats.

Protein-coding genes were predicted using the following approaches: ab initio prediction, homology-based prediction, and transcriptome-based prediction. Around 79 M RNA-seq reads were aligned to the *V. penis* genome using STAR v2.7.3a [33]. The resulting alignment file served as an important support in all three prediction methods. Ab initio gene prediction was performed on the soft repeat-masked assembly with Braker v3.0.3 [34] using default parameters. *Dreissena rostriformis* [35] and *Sinonovacula constricta* [36] were selected and used for homology-based prediction. Protein sequences of *D. rostriformis* and *S. constricta* were downloaded from NCBI and aligned against the assembled genome using MMseqs2 [37] with the parameter “-e 100.0 -s 8.5 --comp-bias-corr 0 --max-seqs 500 --mask 0 --orf-start-mode 1”. The results of homologous alignments were then combined into gene models with the splice site identified using mapped RNA-seq data with GeMoMa v1.9 [38] applying default parameters. Gene predictions were further sorted and filtered separately using the GeMoMa module GAF with default parameters. For the transcriptome-based prediction, the transcriptome of *V. penis* was assembled by both *de novo* and genome-guided approaches using Trinity v2.15.0 [39]. The results were merged and passed to PASA v2.5.2 [40] for gene predictions. In the end, all predictions were combined into consensus coding sequence models using EVidenceModeler v1.1.1 [40], with the weighting of each method as “ab initio 1; homology-based 2, transcriptome-based 8”. Finally, gFACs v1.1.2 [41] was used to remove incomplete gene models. The completeness of the gene set was accessed using BUSCO v5.4.3 [19], protein mode using the lineage dataset metazoa_odb10. The predicted genes were functionally annotated by aligning them to the eggNOG (emapper v1.0.3) databases (2023_06_26) using diamond v0.9.30.131.

### Orthology inference and phylogenetic analysis

Predicted peptide sequences from 30 additional molluscan genomes were used in downstream phylogenetic analyses, representing 23 Bivalvia genomes plus 6 additional conchiferan protein sets used as outgroups (ESM Table S5). Orthology prediction was conducted in OMA v.2.4.1 [42]. Orthologous groups (OGs) were aligned using MAFFT v.7.407 [43] with the L-INS-i algorithm [44] and the command option “-leavegappyregion.” OG alignments were trimmed with GBLOCKS v. 0.91b [45] to cull regions of dubious alignment. An ortholog matrix was built using a custom python script (selectslice.py) [46] to include loci with a minimum of 80% taxa occupancy and was concatenated using Phyutility [47]. Phylogenetic reconstruction was conducted in IQ-Tree v. 2.1.2 [48] using ModelFinder [49] for model selection and ultrafast bootstrap [50] with 1000 replicates. Model finder best fit model based on Akaike Information Criterion (AIC), Corrected AIC, and Bayesian Information Criterion (BIC), LG + F + R7, was used for phylogenetic tree reconstruction in IQ-Tree. Phylogenetic reconstruction and genome assembly were conducted on “Hydra”, the Smithsonian Institution High Performance Cluster (SI/HPC; 10.25572/SIHPC).

### Systematics

The taxonomy of *Verpa penis* has raised some challenges [8, 14, 51] that deserve a short clarification: this species was originally named *Sabella penis*, as Linnaeus interpreted the animal as a tube worm. The genus name was later revised to the genus *Penicillus* Bruguière, 1789 which is taxonomically invalid and a junior homonym of the same name used for polychaetes, an obsolete pre-Linnean name [52]. There is no conflict with a potential alternative use because the polychaete name is not in use; the family name Penicillidae is still valid and correct as a bivalve.

## Results

**Fig. 2:**
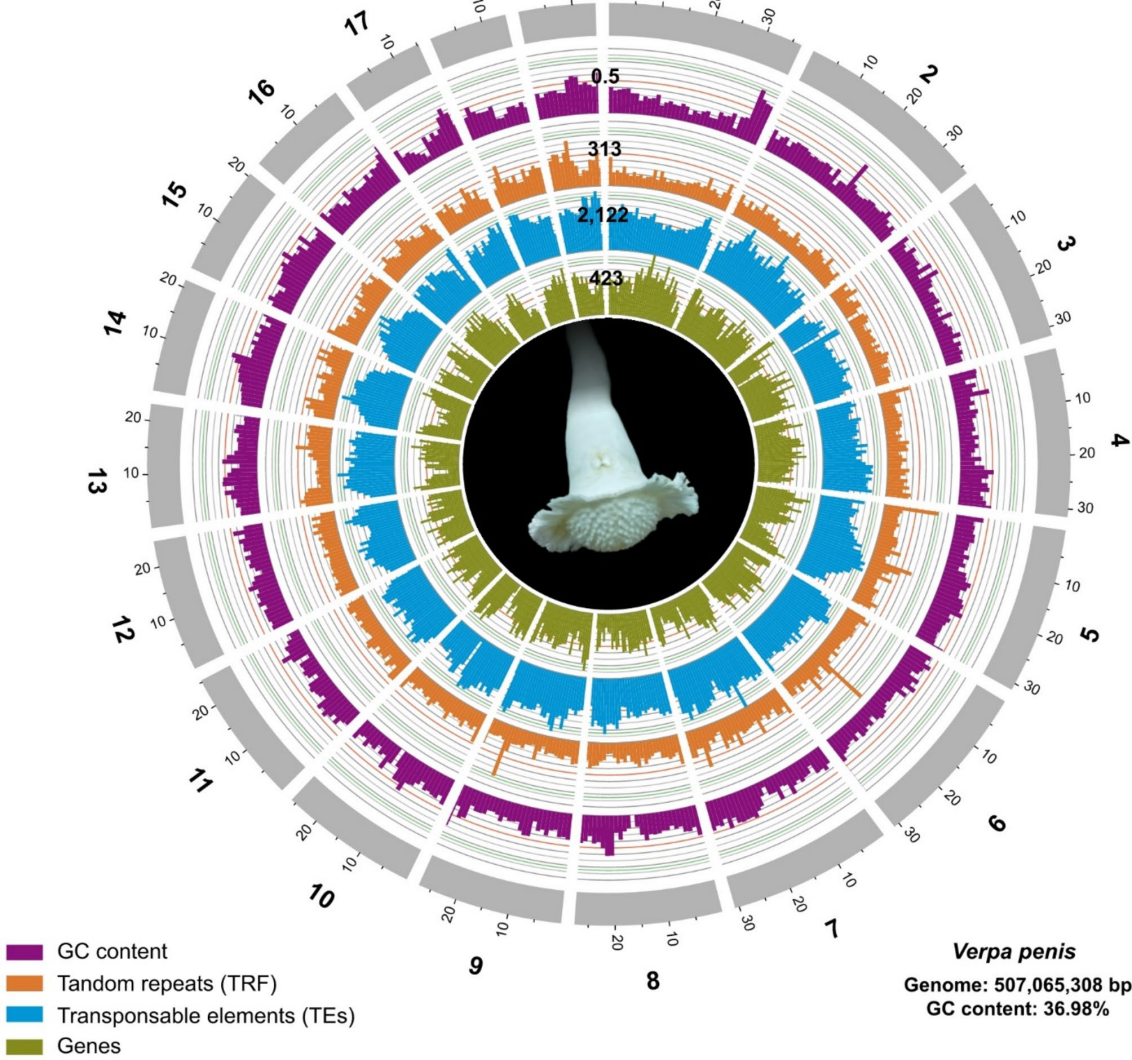
General characteristics of the *V. penis* genome. Tracks from inside to outside correspond to gene number, TEs number, TRF number and GC content in sliding windows of 1 Mb across each of the 19 pseudochromosomes, visualised by Circos v0.69-9. Photo by NLWSW

The genome size of *V. penis* is estimated to be around 487 Mb from the k-mer analysis, and the genomic heterozygosity is estimated to be 0.42% (ESM Fig S1). The whole genome sequencing yielded a total of 17 Gb of HiFi data (ESM Fig S2), which corresponds to a theoretical coverage of 36x. We obtained around 22 Gb (45x) of Hi-C data (145,310,480 reads) to improve the assembly to chromosome level. Finally, we obtained an assembly with a total length of 507 Mb, contig N50 of 5.33 Mb, scaffold N50 of 27.6 Mb, and 96.5% of the sequences anchored onto 19 scaffolds (Fig. 2, ESM Table S2). Contiguity statistics are more than seven-fold higher compared to other bivalves (ESM Table S2). A total of 928 (97.2%) of the BUSCO benchmark set were complete, with only 0.8% duplicated BUSCOs (ESM Table S1); Both contiguous and completeness suggest a high-quality assembly (ESM Fig S3), comparable or exceeding other available mollusc genomes.

Approximately 45.26% of the genome was composed of repetitive elements (38.23% transposons; and 7.03% Tandem repeats; Fig. 2, ESM Table S2). Gene annotation combining evidence from transcripts, homologous proteins, and ab initio prediction resulted in 25,135 predicted protein-coding genes with an average length of 9,113 bp; the average exon/intron length, and the exon and intorn numbers per gene were comparable to other bivalves (ESM Tables S3-S4). Among the predicted protein-coding genes, 76.3% could be functionally annotated; the gene set shows high completeness with 97.9% of complete BUSCOs (ESM Table S5).

**Fig. 3:**
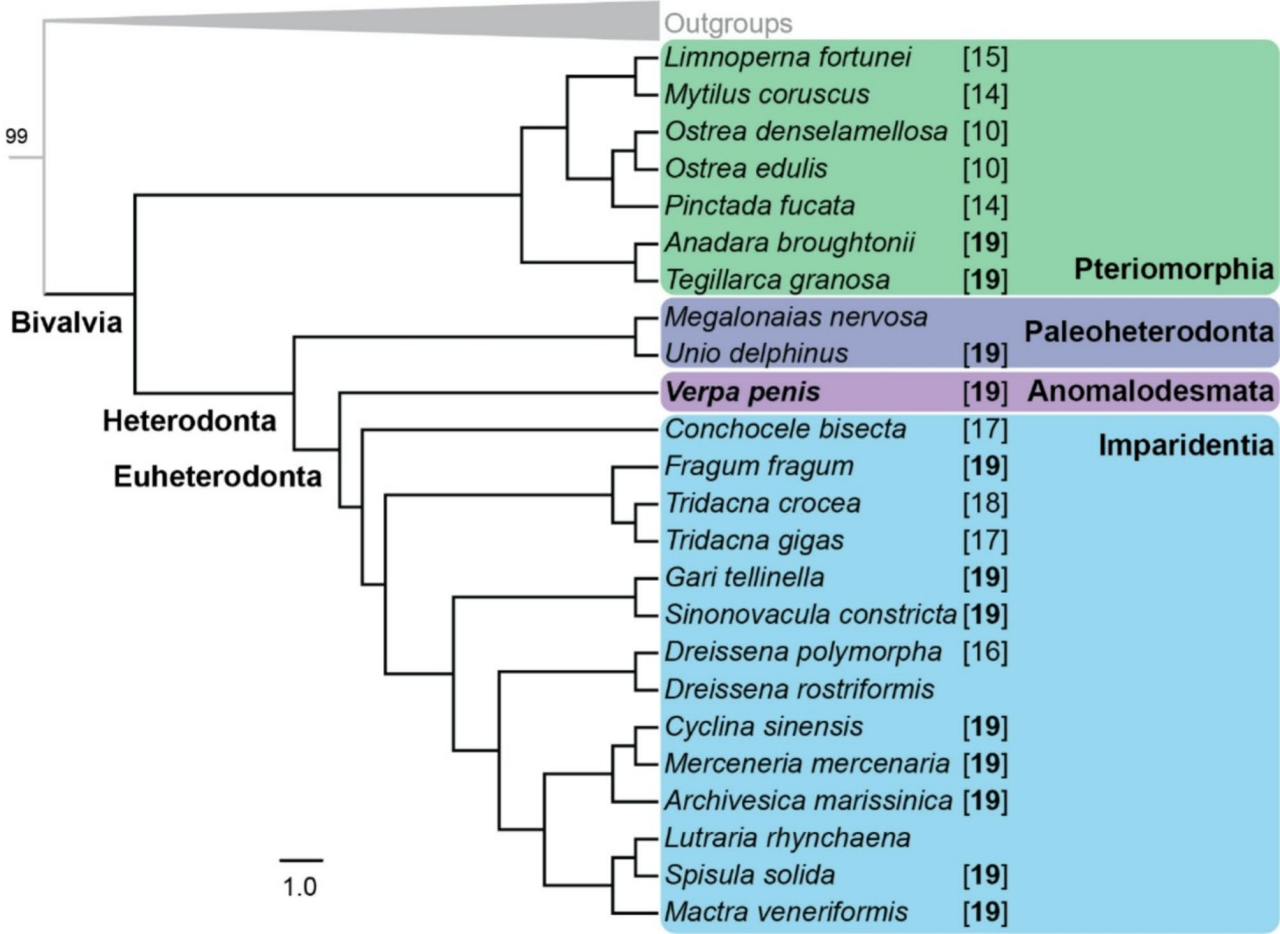
Phylogenetic placement of *Verpa penis*. Maximum likelihood topology generated in IQ-Tree using 80% minimum taxa occupancy matrix (3596 genes, 913028 amino acids). Nodal support values are ML bootstrap proportions < 100. Numbers in square brackets after the taxon names indicate 1 N chromosome numbers

We identified 3595 genes from 23 bivalves and 6 additional conchiferan species. The phylogeny was constructed using 913,028 amino acids with 80% taxa occupancy. We confirms Anomalodesmata, represented by *Verpa*, is sister to Imparidentia (Fig. 3); these two orders together form the clade Euheterodonta [53] with bootstrap value of 100.

## Discussion

### *The* Verpa penis *genome*

Genome resources for molluscs remain scarce for multiple reasons [54]. Bivalves are relatively better sampled than other molluscan clades, but with a disproportionate focus on the clade Pteriomorphia with many species of economic interest (oysters, scallops, mussels and allies) [54, 55]. By contrast, the heterodont bivalves (Euheterodonta, Archiheterodonta, Palaeoheterodonta), comprise a larger fraction of bivalve species richness and broader variety of habitats including freshwater radiations, but lag behind in available genomes.

Early molecular analyses generally found Anomalodesmata inside the historical “Heterodonta” [4, 15, 53], but some proposed alternative placements for Anomalodesmata [56]. Studies that focussed on Anomalodesmata, and later, larger-scale multi-loci and transcriptome analyses identified the families that comprise Euheterodonta [14]. The consistency of this result led to the recognition of Imparidentia as euheterodont bivalves excluding anomalodesmatans [57].

The ancestral genome for Imparidentia had a karyotype of 1 *N* = 19 [58], which is identical to other closely related clades, as known from Paleoheterodonta [59], and now one Anomalodesmata (Fig. 3, ESM Table S5). Most likely this is the ancestral karyotype for all heterodont bivalves. Several lineages show chromosome reductions: in the freshwater invasive zebra mussels, and symbiont-hosting deep-sea *Conchocele* [60] and tropical photosymbiotic *Tridacna* [61, 62]. *Verpa penis* is likewise highly adapted but has an unreduced karyotype.

Genome re-arrangements such as chromosomal inversions and fusion are invoked in explaining adaptive processes [63, 64]. Genome reductions have consistent patterns in the evolution of mammals [65] but this is evidentially not universal [66]. The retention of a full set of chromosomes in *Verpa* does not refute the general idea that species with smaller populations or those in isolated environments might benefit from chromosomal reductions that preserve genetic stability and reduce variation; however, it indicates these are not universal mechanisms.

### Adaptive pathways of the adventitious tube

Evolution employs multiple strategies for molluscs and other animals to maintain position and defend themselves from disturbance. Endobenthic bivalves might increase size and mass, to achieve deeper burial and avoid disturbance [67]. Other adaptations include modifications of the exterior shell sculpture to create a ratchet structure that can resist backsliding [68], or modifying the weight distribution within a shell structure to maintain a position by favouring the heavier side [67, 69].

*Verpa penis* has a relatively smooth shell, and an anterior fringe around the basal plate that is formed of a solid and irregularly ribbed flange. Other tube-forming clavagelloideans have ornamentation including siphonal collars at the top, or anterior tubules at the base. Such structures likely have mechanical functions, to increase surface area or stabilise the position of the animal in the sediment [8]. Tube-forming clavagelloideans are found in various marine sediments, from shelly sand or fine gravel to mud [11, 70, 71]. Species-specific ornamentation may be related to the specific sediment properties of the habitat for each species.

The question of an adaptive driver for the adventitious tube could be approached in context of a conceptual exploration of human-built structures that are optimised for stability in relatively soft sediments. The engineering principles for a stable design in mud or sand incorporate several key strategies: a wide base, low centre of gravity, and suction resistance. A wide base, even for an embedded structure, is crucial to distribute the load and prevent sinking. Sand can shift more easily than mud, so structures in sand may be designed with textured patterns on the lower surface to interlock slightly with the sediment. A low centre of gravity provides stability in the lateral positioning, and prevent tipping. Mud creates a suction force that can pull objects down, making them hard to lift or remove. This is potentially advantageous, when the object is an infaunal bivalve, which, regardless of its lifestyle, requires stability primarily for feeding (e.g., filtering or capturing prey) and reproduction (e.g., releasing gametes), and to avoid being dug up. Suction resistance strategies include cone-shaped or curved bases, and perforations that can break the suction seal when movement is needed. Suction forces are typically weaker in sand because it does not create the same vacuum effect as mud; however, in saturated, fine-grained sand, suction resistance strategies like vent holes would still be beneficial.

The watering-pot shell appears to follow engineering principles similar to those used in suction caisson anchors in mud or sandy seabed installations. Wet sediment can create a suction force that can pull objects down, making them hard to lift or remove, especially if there is a large vertical surface area. An enlarged, cone-shaped or curved base stabilises the structure. These principles mean that suction caissons, or adventitious tubes, once in place, are stable and resistant to being pulled out (via suction) and to lateral forces (such as currents or winds) because of the large surface area in contact with the sediment.

Suction caissons are hollow; the interior is initially filled with water, and then penetration into the sediment is initially aided by using the valve at the bottom of the structure to create a partial vacuum, and the resulting pressure differential effectively but passively drives penetration [72]. For any comparison it is important to bear in mind that the shell is occupied by a living animal that creates a flexible interface within the tube and can voluntarily seal or unseal the basal plate. In watering pot shells, as long as the anterior base of the watering pot is submerged in the sediment, live adult animals can apparently manoeuvre themselves back into normal, buried, vertical position [11, 73, 74]. *Verpa penis* can pump water out of the mantle cavity with the pedal disc, by contracting the its body like the piston and valve of a pump [71], pushing water between the body and the basal plate and out through the holes of the “rose” at the base. Retracting the siphons and sealing the mantle cavity posteriorly creates a temporary partial vacuum within the shell. Pumping water across the perforated basal plate can liquify the surrounding sediment, reducing the surrounding density and resistance [8]. This not only allows the bivalve to sink into the substrate but would also aid in maintaining its position within the sediment.

Anatomical modifications observed in most adult clavagelloids—such as a reduced foot, degenerated adductor muscles, an expanded mantle cavity and even the absence of byssus threads [75]—may support the plausibility of this suction anchor theory. The same suction strategy could also provide increased sediment-stability to clavagelloids during certain stages of development after the loss of the byssus thread [8]; however, scant information is known about the developmental process of clavagelloids.

Over time, the adventitious tube has taken on another major role of the shell: acting as an exoskeletal attachment site for the reduced musculature [11]. The whole watering pot structure—including the adventitious tube as an intrinsic extension—enables efficient burrowing, stabilisation, and resistance to removal, exploiting the natural suction property of soft marine sediments.

The present study benefitted from an unusually high number of specimens found in a single collecting event, where we were able to find six individuals—noting that there are only four prior published reports of clavagelloids observed alive [51, 71, 73, 74]. Conditions during that event were abnormal, with heavy rain producing a freshwater layer on top of a tidally exposed shoal, and the animals were found by tactile searching rather than visually. This opportunistic collection may reflect circumstance where the sediment was liquified and shifting around the animals such that they could not reposition themselves vertically.

It is clear that this species is rare, as typical of all Clavagelloidea [8], and highly vulnerable to environmental disturbance that would shift sediment profiles. The population in Johor, Malaysia, is potentially threatened by anthropogenic activities through active land reclamation, and geographically adjacent to a population in Singapore that was recently rediscovered after being reported as locally extinct [51].

## Conclusions

In evolutionary biology we frequently seek to explain adaptations, or at least classify them in terms of determinism (inevitable consequence of functional need) or contingency (many different ways to solve the same problem). The convergence of adventitious tubes in clavagelloidean bivalves is apparently a deterministic outcome of optimising stability in soft sediments, and may parallel the engineering principles of suction anchors. The genome of *Verpa penis* demonstrates that such remarkable modification in body plan does not correlate to significant changes in genome architecture.

## Electronic supplementary material

Below is the link to the electronic supplementary material.

Supplementary Material 1

## Data Availability

The whole genome sequencing data and novel genome assembly of Verpa penis are available via the National Center for Biotechnology Information (NCBI) under the PRJ number PRJNA1120794. The transposon-masked genome, the gene annotation files and the corresponding coding and protein sequences, and the transposon annotation files are stored in the Verpa penis genome project at figshare: https://figshare.com/account/home#/projects/243845Files for the phylogenetic analyses are available via https://dataportal.senckenberg.de/dataset/verpaSpecimens are deposited in the University Putra Malaysia Marine Collection (UPMMC), Port Dickson, Malaysia.
