# Supplementary material for: Genome of the enigmatic watering-pot shell and morphological adaptations for anchoring in sediment
Source: BMC Genomics. 2025 May 9;26:460. doi: 10.1186/s12864-025-11622-w (PMC12063269; doi:10.1186/s12864-025-11622-w)
Supplement: Supplementary file 1 — Supplementary Material 1 [file 12864_2025_11622_MOESM1_ESM.docx]

**Electronic Supplementary Materials**

Additional genome measures (Figures S1-S3, Tables S1-S4) are summarised below for *Verpa penis*, as well as the source data for the additional species used in the phylogenetic reconstruction (Table S5).


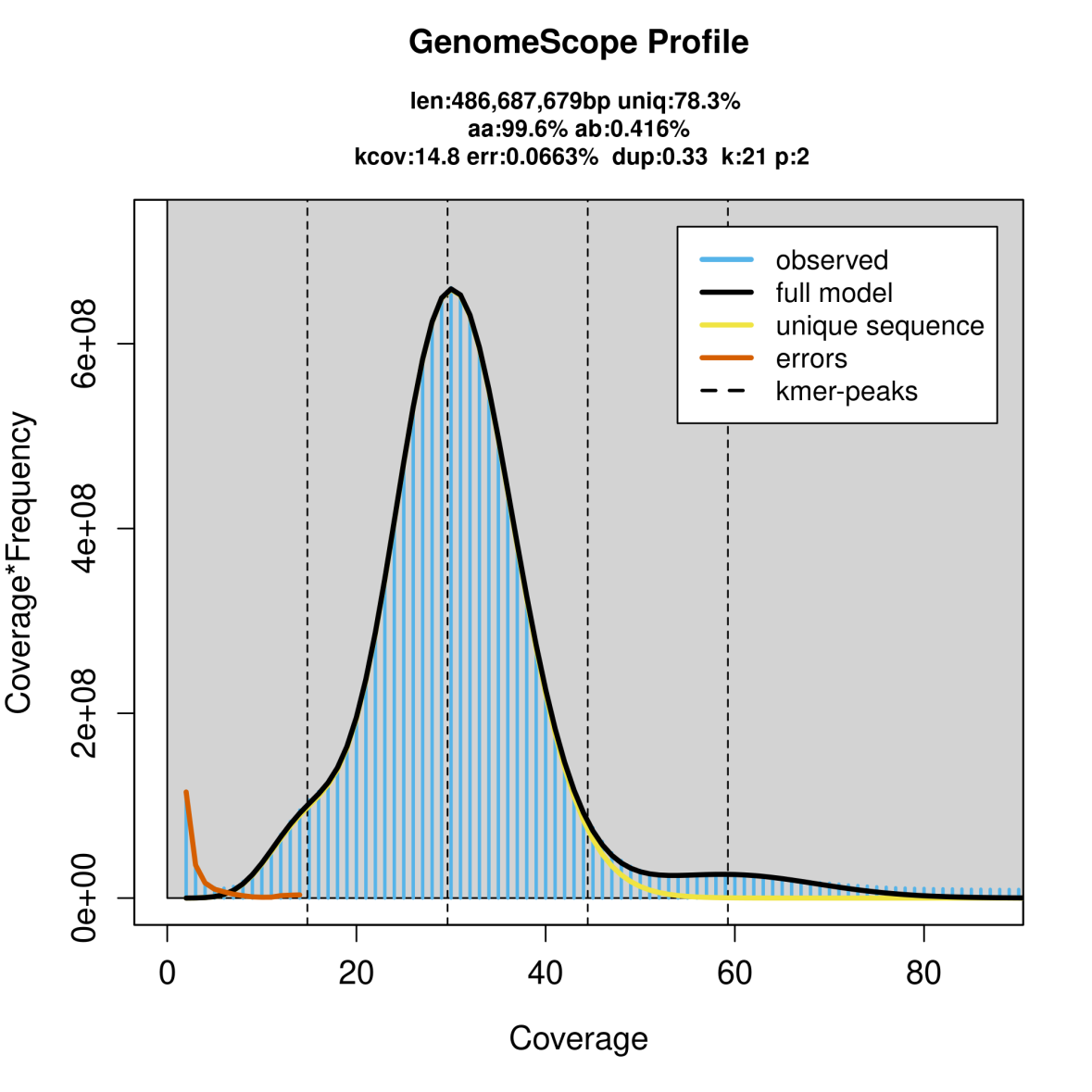


**Fig S1.** Estimation of genome features based on the distribution of 21-mer frequency.


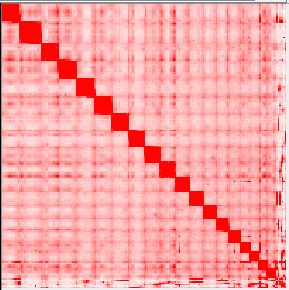


**Fig S2.** Hi-C chromosome contact maps. Each block represents a Hi-C contact between two genomic loci within a 1 Mb window. The darker the shade of a block, the higher the contact intensity.


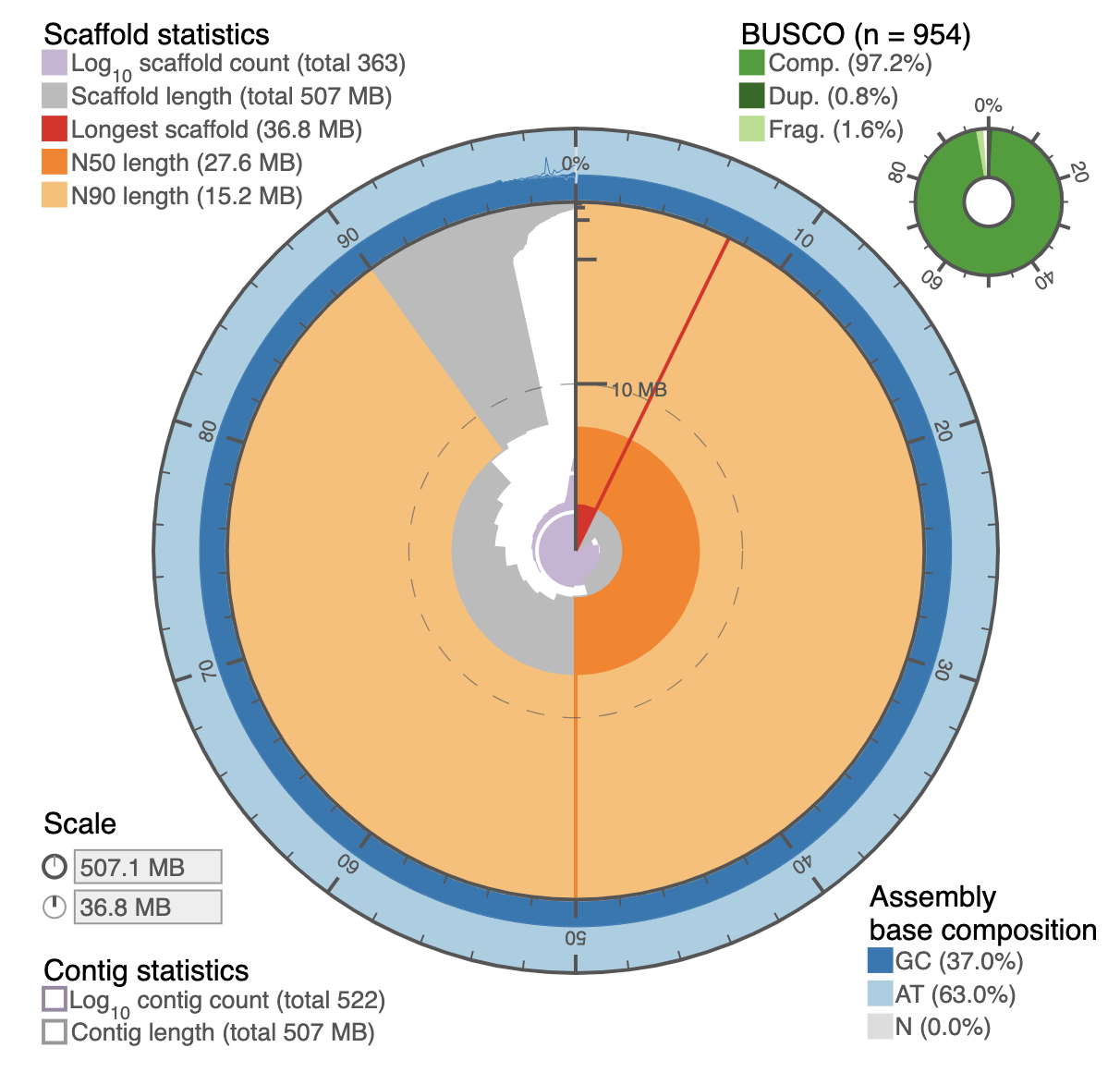


**Figure S3** Snail plot summarizing the genome statistics of *Verpa penis*. Snail plots were generated using BlobToolKit v 3.3.10 (Challis et al., 2020). The distribution of scaffold lengths is shown in dark gray with the plot radius scaled to the longest sequence present in the assembly (36.8Mb, shown in red). Statistics of note shown here include the genome N50: 27.6 Mb (dark orange), N90: 15.2Mb (light orange),and base composition (percentage of GC in dark blue, AT in light blue, and N in light gray). BUSCO results summarizing complete, duplicated and fragmented using the Metazoa_odb10 dataset are displayed in top right (in shades of green).

**Table S1.** Statistics of the final assembly at different assemble stages.

| **Characteristic** | **Value** |
| --- | --- |
| Total Contig length (bp) | 507,038,511 (522) |
| Contig N50 (bp) | 5,325,940 (28) |
| Longest Contig (bp) | 16,098,946 |
| Total scaffold length (bp) | 507066948 (636) |
| Scaffold N50(bp) | 27,570,495 (8) |
| Longest Scaffold (bp) | 36,765,564 |
| N's per 100 kbp | 5.61 |
| Complete BUSCOs | 97.2% (928) |
| Complete and single-copy BUSCOs | 96.4% (920) |
| Complete and duplicated BUSCOs | 0.8% (8) |
| Fragmented BUSCOs (F) | 1.6% (15) |
| Missing BUSCOs (M) | 1.2% (11) |

Table S2. Identified repeat classes in the *V. penis* genome.

| **Type** | **Repeat Size(bp)** | **% of Genome** |
| --- | --- | --- |
| Tandem repeats | 35,671,495 | 7.03 |
| Transposable elements (total) | 193,845,983 | 38.23 |
| DNA | 46,038,611 | 1.03 |
| LINE | 13,410,503 | 2.64 |
| LTR | 7,525,767 | 1.48 |
| Low | 875,480 | 0.17 |
| PLE | 643,942 | 0.13 |
| RC | 17,276,857 | 3.41 |
| Retroposon | 1,216 | 0.00 |
| SINE | 25,028,422 | 4.94 |
| Satellite | 126,310 | 0.02 |
| Unknown | 95,656,288 | 18.86 |
| scRNA | 835 | 0.00 |
| srpRNA | 3,291 | 0.00 |

Table S3. Statistics of predicted protein-coding genes in the *V. penis* genome.

| **Gene set** | **Total Genes Predicted** | **Average Transcript Length(bp)** | **Average CDS Length(bp)** | **Average Exon per Gene** | **Average Exon Length(bp)** | **Average Intron Length(bp)** |
| --- | --- | --- | --- | --- | --- | --- |
| *de novo* | 29,797 | 9168.03 | 1578.68 | 7.94 | 198.94 | 1094.31 |
| *Dreissena rostriformis* | 17,494 | 4,982.25 | 1,194.25 | 4.64 | 257.61 | 1,041.83 |
| *Sinonovacula constricta* | 15,305 | 6859.31 | 1,386.41 | 6.09 | 227.75 | 1,075.77 |
| mRNA | 102,143 | 5,095.79 | 1,103.53 | 2.99 | 369.01 | 2,005.64 |
| EVM | 25,174 | 9,103.73 | 1,534.29 | 7.47 | 205.48 | 1,170.51 |
| **Final set** | **25,135** | **9,112.83** | **1,535.27** | **7.47** | **205.41** | **1,170.40** |

**Table 4.** Gene set completeness measured by Benchmarking Universal Single-Copy Orthologs (BUSCO). Note: Busco was run in the “ -m protein” mode to access the gene set completeness, this is different form the genome completeness estimation in Table S1.

|  | **Number of genes** | **Percent Completeness (%)** |
| --- | --- | --- |
| Complete BUSCOs | 934 | 97.9 |
| Complete Single-copy BUSCOs | 927 | 97.2 |
| Complete Duplicated BUSCOs | 7 | 0.7 |
| Fragmented BUSCOs | 9 | 0.9 |
| Missing BUSCOs | 11 | 1.2 |

**Table S4.** NCBI GenBank Accessions from publicly available data and newly sequenced *Verpa penis* (in bold) used for phylogenetic reconstruction.

| **Class** | **Species name** | | **Assembly Accession** | **Sources** |
| --- | --- | --- | --- | --- |
| Scaphopoda | *Pictodentalium vernedei* | | GCA_031216915.1 | Song et al. 2023 |
|  | *Siphonodentalium dalli* | | GCA_032622095.1 | Song et al. 2023 |
| Gastropoda | *Chrysomallon squamiferum* | | GCA_012295275.1 | Sun et al. 2020 |
|  | *Lottia gigantea* | | GCF_000327385.1 | Simakov et al. 2013 |
|  | *Haliotis rubra* | | GCF_003918875.1 | Gan et al. 2019 |
|  | *Haliotis rufescens* | | GCF_023055435.1 | Griffiths et al. 2022 |
| Bivalvia | *Anadara broughtonii* | | <http://dx.doi.org/10.5524/100607> | Biao et al. 2019 |
|  | *Archivesica marissinica* | | GCA_014843695.1 | Ip et al. 2021 |
|  | *Cyclina sinensis* | | GCA_012932295.1 | Wei et al. 2020 |
|  | *Conchocele bisecta* | | GCA_029237695.1 | Guo et al. 2023 |
|  | *Dreissena rostriformis* | | GCA_007657795.1 | Calcino et al. 2019 |
|  | *Dreissena polymorpha* | | GCA_020536995.1 | McCartney et al. 2022 |
|  | *Fragum fragum* | | GCA_946902895.1 | Li et al. 2024a |
|  | *Gari tellinella* | | GCA_922989275.2 | Holmes et al. 2022 |
|  | *Lutraria rhynchaena* | | GCA_008271625.1 | Thai et al. 2019 |
|  | *Limnoperna fortunei* | | GCA_944474755.1 | Ferreira et al. 2023 |
|  | *Mactra veneriformis* | | GCA_025267735.1 | Sun et al. 2022 |
|  | *Megalonaias nervosa* | | GCA_016617855.1 | Gomes-Dos-Santos et al. 2023a |
|  | *Merceneria mercenaria* | | GCA_021730395.1 | Farhat et al. 2022 |
|  | *Mytilus coruscus* | | GCA_017311375.1 | Yang et al. 2021 |
|  | *Ostrea denselamellosa* | | GCA_024699665.1 | Dong et al. 2023 |
|  | *Ostrea edulis* | | GCF_947568905.1 | Boutet et al. 2022 |
|  | *Pinctada fucata* | | GCA_028142955.1 | Takeuchi et al. 2022 |
| Bivalvia | *Unio delphinus* | | GCA_029339505.1 | Gomes-Dos-Santos et al. 2023b |
|  | *Sinonovacula constricta* | | GCA_007844125.1 | Ran et al. 2019 |
|  | *Spisula solida* | | GCA_947247005.1 | Holmes et al. 2023 |
|  | *Tegillarca granosa* | | GCA_029721355.1 | Bao et al. 2021 |
|  | *Tridacna crocea* | | GCA_943736015.1 | Li et al. 2023 |
|  | *Tridacna gigas* | | GCA_945859785.2 | Li et al. 2024b |
|  | ***Verpa penis*** | | **JBEUMR000000000** | **This study.** |
|  |  |  | |  |
